# The CD8 T Cell-Epstein-Barr Virus-B Cell Trialogue: A Central Issue in Multiple Sclerosis Pathogenesis

**DOI:** 10.3389/fimmu.2021.665718

**Published:** 2021-07-07

**Authors:** Caterina Veroni, Francesca Aloisi

**Affiliations:** Department of Neuroscience, Istituto Superiore di Sanità, Rome, Italy

**Keywords:** multiple sclerosis, CD8 T cells, B cells, Epstein-Barr virus (EBV), anti-EBV immunity

## Abstract

The cause and the pathogenic mechanisms leading to multiple sclerosis (MS), a chronic inflammatory disease of the central nervous system (CNS), are still under scrutiny. During the last decade, awareness has increased that multiple genetic and environmental factors act in concert to modulate MS risk. Likewise, the landscape of cells of the adaptive immune system that are believed to play a role in MS immunopathogenesis has expanded by including not only CD4 T helper cells but also cytotoxic CD8 T cells and B cells. Once the key cellular players are identified, the main challenge is to define precisely how they act and interact to induce neuroinflammation and the neurodegenerative cascade in MS. CD8 T cells have been implicated in MS pathogenesis since the 80’s when it was shown that CD8 T cells predominate in MS brain lesions. Interest in the role of CD8 T cells in MS was revived in 2000 and the years thereafter by studies showing that CNS-recruited CD8 T cells are clonally expanded and have a memory effector phenotype indicating *in situ* antigen-driven reactivation. The association of certain MHC class I alleles with MS genetic risk implicates CD8 T cells in disease pathogenesis. Moreover, experimental studies have highlighted the detrimental effects of CD8 T cell activation on neural cells. While the antigens responsible for T cell recruitment and activation in the CNS remain elusive, the high efficacy of B-cell depleting drugs in MS and a growing number of studies implicate B cells and Epstein-Barr virus (EBV), a B-lymphotropic herpesvirus that is strongly associated with MS, in the activation of pathogenic T cells. This article reviews the results of human studies that have contributed to elucidate the role of CD8 T cells in MS immunopathogenesis, and discusses them in light of current understanding of autoreactivity, B-cell and EBV involvement in MS, and mechanism of action of different MS treatments. Based on the available evidences, an immunopathological model of MS is proposed that entails a persistent EBV infection of CNS-infiltrating B cells as the target of a dysregulated cytotoxic CD8 T cell response causing CNS tissue damage.

## Introduction

Multiple sclerosis (MS) is a chronic inflammatory disease of the central nervous system (CNS) and one of the world’s most common neurological disorders. The disease is characterized by multifocal inflammatory lesions causing demyelination and neurodegeneration in the brain and spinal cord and leading to the progressive loss of motor, sensory and cognitive functions (1). MS usually has a relapsing remitting course at onset, followed by a secondary progressive phase with gradual worsening of the neurological symptoms; in a minority of patients the disease has a progressive onset. Drugs approved for relapsing remitting MS can reduce clinical relapses and inflammatory disease activity and delay to some extent the progression of neurological deficits (2, 3). However, a definitive cure for MS is still missing.

The etiology of MS involves multiple genetic risk factors acting in concert with environmental exposures (4). Genome-wide association studies (GWAS), over the last decade, have identified more than 230 loci associated with MS susceptibility (5–9). The strongest genetic association for MS maps to the class II region of the human leukocyte antigen (HLA) gene cluster in chromosome 6p21. The ubiquitous herpesvirus Epstein-Barr virus (EBV), cigarette smoking, and vitamin D deficiency are the environmental factors that show the most consistent association with MS (4, 10). Among these, EBV has the strongest impact on the immune system due to its ability to establish a life-long latent infection in B cells, reactivate periodically and induce a very effective immune response that is essential to counteract the virus lymphoproliferative potential (11, 12). The epidemiological data associating EBV infection to MS, the main features of EBV biology and immunology, the compatibility between EBV and MS disease biology, and the potential mechanisms linking EBV to CNS inflammation are discussed in the following sections and are summarized in **Table 1**.

**Table 1:** EBV intersection with MS.

EBV features	Brief description	Compatibility with MS
EBV epidemiology	Ubiquitous DNA herpesvirus that infects about 90% of the global population. EBV infection is mostly asymptomatic in childhood but primary exposure during adolescence or adulthood frequently causes infectious mononucleosis (13).	Previous exposure to EBV is required, though not sufficient, to develop MS (14). Infectious mononucleosis (15) and high anti-EBNA-1 IgG titers (16–18) increase the risk of MS. There are similarities between the epidemiology of infectious mononucleosis and that of MS, including higher socioeconomic status, latitude gradient, earlier onset in women than in men (19, 20).
EBV biology	EBV is transmitted through saliva, infects mainly B cells and epithelial cells and establishes a life-long latent infection in memory B cells (11). The virus first establishes a lytic infection in the oropharyngeal epithelial cells and then switches to latent infection of B cells in the local lymphoid tissue (tonsils). Initially, EBV establishes a growth transforming latent infection of B cells (latency III or growth program) leading to proliferation of the infected cells; at this stage of infection, all EBV latent proteins [EBV nuclear antigen (EBNA) 1, 2, 3A, 3B, 3C, and -LP; latent membrane protein 1 (LMP1) and LMP2], several small noncoding RNAs and micro-RNAs, and the EBV-encoded small RNAs (EBER1, EBER2) are expressed. EBV infected B lymphoblasts receive survival and activating signals from LMP1 and LMP2A, that mimic B cell receptor stimulation by cognate antigen and T cell help through CD40 signalling, respectively. EBV then enters a more restricted form of latency where only EBNA1, LMP1 and LMP2 are expressed (latency II program), whereas only EBNA1, that is required for replication of the episomal EBV genome, is expressed in latency I. Viral persistence and avoidance of immune surveillance are achieved as the result of downregulation of all EBV gene products in EBV infected resting memory B cells that enter the blood circulation (latency 0). Occasional viral reactivations occur in the tonsils, where recirculating EBV infected B cells differentiate into plasma cells leading to production of viral particles that can infect new B cells locally and also result in viral shedding into saliva. The replicative viral cycle involves the sequential expression of a large array of immediate early, early and late lytic genes (>80) encoding proteins that are implicated in the production of new viral particles and (some of them) also in immune evasion.	The B-cell growth promoting properties of EBV could explain the expansion and differentiation of B cells in the CNS of MS patients throughout the disease.
		Persistent, treatment resistant intrathecal B-cell activation and immunoglobulin synthesis are a characteristic of MS (21, 22). More than 90% MS patients have oligoclonal IgG in the CSF. Oligoclonal IgG in the CSF are typically found in acute CNS infections, but their specificity in MS is unknown although recognition of EBV proteins has been reported (23–26). Polyspecific immunoglobulins recognizing common viruses (rubella, measles, varicella zoster, less commonly EBV) are synthesized in the CNS of nearly 80% of MS patients, and are believed to reflect non-specific bystander B-cell activation (22).
		B cells and plasmablasts/plasma cells, usually absent in the normal CSF, are found in the CSF of MS patients and their number correlates with inflammation, blood-brain barrier breakdown and intrathecal Ig synthesis (27–29). B cell receptor repertoire analysis in MS patients has revealed presence of clonally expanded B cells in CSF, brain tissue and meninges indicative of local antigenic stimulation (21, 30) as well as trafficking of activated B cells between the CNS, draining cervical lymph nodes and peripheral blood (29, 31, 32).
		B cells and plasma cells are found in CNS tissue in early and chronic MS stages (33–35), mainly within the perivascular space of intraparenchymal blood vessels and the subarachnoid space in the meninges. Large B-cell aggregates resembling B-cell follicles and containing stromal cells, proliferating B cells and plasma cells are present in the meninges of patients with progressive MS (36, 37). The pathogenic role of these structures is indicated by their association with increased cortical damage and MS disease severity (37–39).
EBV immunology	Continuous immune surveillance is essential to maintain virus-host homeostasis throughout the host’s life. Following primary infection, the rapid antibody response to EBNA2 and EBV lytic proteins, like the virus capsid antigen (VCA), is important to control the virus, and is followed by a slow increase of the neutralizing antibody response (mainly towards the major envelope EBV protein gp350) and a delayed EBNA1 IgG response. Most studies of the T-cell response to EBV during primary infection have been carried out in people with symptoms of infectious mononucleosis. Early control of EBV infection is associated with expansion of innate immune cells, mainly NK cells (40), IFNγ producing cytolytic CD8 T cells and, to a lesser extent, CD4 T cells. While CD4 T cells recognize a broad range of EBV latent and lytic proteins, CD8 T cells recognize mainly EBV lytic proteins (41). During persistent infection, memory CD4 T cells are present at low frequency, recognize mainly latent EBV proteins, do not express activation markers and belong to both the central memory and effector memory subsets. Compared to the CD4 T cell response, the EBV-specific CD8 T cell response in the blood of healthy carriers is much greater and skewed towards immunodominant EBV latent (EBNA3A, 3B, 3C) and immediate early (BZLF1, BRLF1) and early (BMRF1, BMLF1) lytic EBV proteins, the response to lytic proteins occurring at higher frequency. Circulating EBV-specific CD8 T cells are resting, antigen experienced T cells that exhibit potent effector functions, including cytotoxicity and cytokine (IFNγ, TNF) production, upon antigen challenge. Due to an efficient immune control, rare EBV infected memory B cells are present in the peripheral blood (1-50 in 10^6^ B cells) and lymphoid tissue of healthy carriers. Cytotoxic lymphocytes, including NK cells and EBV-specific CD8 T cells, have a key role in limiting EBV lytic replication and preventing EBV-driven pathologies (40). The EBV-host balance is perturbed when genetic factors or other factors, such as immunosuppression, alter or abolish the cytotoxic control of EBV.	Altered humoral and cell-mediated immune responses to EBV in MS patients suggest EBV dysregulation/inadequate virus control. MS patients are 100% EBV seropositive and have higher serum titers of EBNA1 IgG and anti-VCA IgG compared to healthy individuals (10, 42). An increase in EBNA-1 IgG is detectable about 5 years up to 20 years before the first disease symptoms (17, 18). Higher EBNA-1 IgG titers have been associated with conversion to definite MS in patients with a clinically isolated syndrome (43) and with cortical atrophy and lesion burden in patients with MS (44). The association between anti-EBV antibodies and clinical or radiological MS disease activity is controversial (45–48). Regarding EBV-specific T cell responses, the CD4 T cell response to EBNA-1, but not CMV epitopes, is increased in the peripheral blood of MS patients compared to control subjects (49) and CD4 T cells recognizing EBNA-1 or EBV transformed B-cell lines have been detected in the CSF of MS patients (50–52). The EBV-specific CD8 T cell response is increased, reduced or unchanged in MS patients compared to control subjects depending on disease activity and duration, and on the T cell specificities investigated. Higher frequencies of EBV-specific T cells are found in the peripheral blood at disease onset and during relapses (53–56). The frequency and functionality of EBV-specific CD8 T cells decreases with increasing disease duration (54, 55, 57, 58), suggesting T cell exhaustion. EBV-specific CD8 T cells, recognizing mainly lytic EBV proteins, are enriched in the CSF (59–61) and brain tissue (62, 63) of MS patients. Defects in NK cell function are present in MS (64). Expansion of CD8+ NK cells in the blood of MS patients has been associated with a favourable clinical outcome (65), raising the possibility that this cell subset is involved in the control of EBV infection.
EBV pathogenic potential	EBV is etiologically linked to a wide range of human malignancies, including B-cell malignancies, like Hodgkin’s lymphoma, Burkitt’s lymphoma, diffuse large B cell lymphoma and post-transplant B-lymphoproliferative disease, NK/T cell lymphoma and nasopharyngeal carcinoma (12).	The mechanisms linking EBV infection to MS pathology remain elusive. Several hypotheses have been proposed, each calling for further studies:
	EBV is also the etiological agent of immunopathologic diseases that are caused by an excessive immune response towards uncontrolled EBV infection, like infectious mononucleosis, a self limiting lymphoproliferative disease, and chronic active EBV infection, a very serious condition with persistence of infectious mononucleosis-like symptoms and hemophagocytic lymphohistiocytosis. In infectious mononucleosis a large proportion of memory B cells are infected with EBV (up to 50% in the peripheral blood) causing the large expansions of NK cells and highly activated EBV-specific T cells, which are predominantly CD8 T cells specific for EBV lytic proteins, and release of pro-inflammatory cytokines, including high amounts of IFNγ. This aggressive cytotoxic immune response correlates with the severity of infectious mononucleosis symptoms, including fever, lymphadenopathy and prolonged fatigue (13). Several rare primary immunodeficiencies affecting NK and T cell function result in failure to control EBV infection predisposing to EBV-associated pathologies, like B-cell lymphomas, fulminant infectious mononucleosis and hemophagocytic lymphohistiocytosis (76).	▪ According to Pender (66), EBV infection may rescue autoreactive B cells producing antibodies to CNS proteins that migrate into the CNS and provide costimulatory signals for CNS autoreactive CD4 T cells. To date, MS-associated pathogenic autoantibodies remain undefined (67), and there is no evidence that EBV favors survival of autoreactive B cells (68). ▪ The molecular mimicry hypothesis is supported by several studies showing that antibodies (69–71) and CD4 T cells (72–75) from MS patients cross react with peptides from EBV proteins and peptides from myelin or other proteins expressed in the CNS. Crossreactive antibodies and CD4 T cells can be found also in healthy individuals, albeit at lower frequency. The pathogenicity of antibodies and T cells recognizing candidate MS autoantigens remains to be demonstrated. ▪ The mistaken self hypothesis proposes that EBV infection induces expression of the small heat shock protein alpha B-crystallin in B cells; HLA-DR-restricted presentation of alpha B-crystallin activates pathogenic CD4 T cells recognizing stress-induced alpha B-crystallin in glial cells in MS brain lesions (77). There is no evidence for increased T cell responses towards alpha B-crystallin in MS patients (78) or recognition of this protein by CSF-infiltrating T cells from MS patients (51, 61). ▪ EBV-driven immunopathology entails that bystander CNS tissue damage is caused by a cytotoxic T cell response towards a persistent, reactivated EBV infection in the CNS. In support of this hypothesis are the findings that CD8 T cells are activated and preferentially expand in the CNS of MS patients (79–83), and that EBV-specific CD8 T cells are selectively enriched in the CSF and brain tissue of MS patients (59–63). Presence of EBV latently infected B cells and EBV reactivation in the MS brain is highly debated (see text).

At the CNS level, MS is characterized by immune abnormalities that include B-cell activation manifesting as persistent intrathecal immunoglobulin (Ig) synthesis and oligoclonal IgG bands, moderate pleiocytosis and increased levels of pro-inflammatory cytokines and chemokines in the cerebrospinal fluid (CSF) (1). Inflammatory cells (T cells, B cells, plasma cells and myeloid cells) accumulate mainly within the perivascular space of post-capillary venules in the white matter and, to a lesser extent, gray matter, and in the subarachnoid space within the meninges (33). Activation of microglia, the CNS resident innate immune cells, is another prominent feature of CNS inflammation in MS, contributing both to tissue injury and to the healing response (84).

For nearly 40 years, the studies addressing the identity and antigenic specificity of T cells migrating and becoming activated in the CNS have focussed on CD4 T helper (Th) cells and Th subsets (Th1, Th17, Th1/Th17) as executors of putative, still elusive, autoimmune responses towards myelin proteins (85). Quietly, but steadily, evidence has grown for an important role of CD8 T cells and B cells in MS immunopathogenesis. These knowledge advancements have been mostly driven by two sets of data: i) new evidences pointing to EBV as a possible causative agent in MS (16, 17) and a biologically plausible trigger or driver of the recurrent, highly destructive CNS-targeted immune response (49, 66, 86, 87) brought more attention on CD8 T cells as these cells play a key role in antiviral immunity and dominate the CNS immune infiltrate in MS (79); ii) the discovery of B-cell follicle-like structures in the brain meninges and the demonstration of their association with disease severity and cortical damage in patients with progressive MS (36, 37, 88), together with the high efficacy of B-cell depleting monoclonal antibodies in suppressing MS disease activity (89) have fostered investigations on B-lineage cells beyond their role as antibody producing cells (90).

Once the key immune cell players are identified, the main challenge is to define precisely how they act and interact to promote chronic CNS inflammation and the downstream neurodegenerative cascade in MS. This article places a special emphasis on the human genetic, neuropathologic, immunologic and molecular studies that have contributed to elucidate the role of CD8 T cells in MS pathogenesis. The available data are discussed in the context of current understanding of autoimmunity, B-cell and EBV involvement in MS, and of the impact of approved MS treatments on T cells and B cells. By wrapping up this information, a model of MS pathogenesis is proposed that entails defective immune control of EBV in susceptible individuals as an early event leading to establishment of a persistent EBV infection in the CNS and chronic activation of an EBV-specific cytotoxic T cell response as the main determinant of CNS tissue damage. This model challenges the paradigm that MS is an autoimmune disease, paving the way towards new ways to treat and prevent the disease.

## MS Risk Genes Linked to CD8 T Cell and B Cell Function

The strongest genetic factor determining MS risk is the HLA-DRB1*15 group of alleles implicating CD4 T helper cells in disease pathogenesis (91). The mechanisms by which the HLA-DRB1*15 haplotype affects MS susceptibility remain to be precisely defined but may include HLA-DRB1*15 hypomethylation causing changes in HLA-DRB1*15 expression on antigen presenting cells (APC) (92) and presentation of self peptides by APC that activate CD4 T cells recognizing antigens expressed in the CNS (72, 93, 94).

MHC class I alleles are also associated with MS suggesting CD8 T cell involvement. HLA-A*0301 increases the risk of MS by about two-fold (95) while HLA-A*0201 confers protection from the disease, halving the risk associated with HLA-DRB1*15 (5, 95). A study in humanized transgenic mice expressing a myelin-specific T cell receptor derived from a CD8 T cell clone from an individual patient along with HLA-A*0301 or HLA-A*0201 showed an opposite effect of these alleles on the development of experimental autoimmune encephalomyelitis, suggesting a potential link between MS genetic risk and CD8 T cell-mediated autoimmune demyelination (96). Of interest, HLA-A*0201 and another MS protective MHC class I allele, HLA-B*44, were recently associated with increased levels of the type 1 interferon (IFN) receptor subunit IFNAR2 in B and T cells and a reduced response to type I IFN stimulation in both cell types (97). These findings suggest that the protective effect of these MHC class I alleles in MS might be mediated by modulation of a key pathway in antiviral immunity.

The association between EBV and MS is the best documented pathogen-chronic disease association and is supported by a large body of seroepidemiological data pointing to an altered virus-host balance before and during the disease (10, 19) (**Table 1**). There is solid evidence that EBV infection is necessary, though not sufficient, for MS development (14, 98, 99) and that the risk of developing MS is increased in individuals with a history of infectious mononucleosis (15). Higher levels of antibodies to EBV, mainly anti-EBV nuclear antigen-1 (EBNA-1) IgG, but not to other viruses, are present in MS patients compared to healthy subjects and predispose to MS development (10). These evidences have led to investigate whether HLA genes influence MS genetic risk through the control of EBV infection. A suboptimal antibody and cytotoxic response to EBV during primary infection could result in a higher viral load, altering the virus-host immune system balance and increasing the chance for the virus to activate pathologic processes potentially leading to MS, as summarized in **Table 1** and discussed below.

Because cytotoxic CD8 T cells are predominantly involved in the killing of virus infected cells and CD4 T cell help is required for CD8 T cell priming, maintenance of CD8 T cell memory and prevention of exhaustion (100, 101), it is likely that both class I- and class II-restricted antigen presentation is involved in EBV control. An interaction between EBV infection and HLA genes associated with MS risk is suggested by the finding that HLA-DRB1*15 carriers with higher anti-EBNA-1 antibody titers have a markedly elevated risk of MS (102–104), which is increased further in the absence of the protective allele HLA-A*0201 (103). MS risk-associated non-MHC genes involved in B and T cell activation, like MYB, CARD11, and CLEC16A were also found associated with higher EBNA-1 IgG levels in MS patients (104).

A recent study in humanized mice (NOD-scid IL2 receptor γ-chain-deficient mice reconstituted with human immune system components) has shown less efficient recognition of EBV-transformed B cell lines by HLA-DR15-restricted CD4 T cell clones, increased CD8 T cell expansion and activation, and higher EBV DNA load in HLA-DR15 donor-reconstituted mice after EBV infection (105). In the same study, HLA-DR15-restricted CD4 T cell clones also recognized myelin basic protein (MBP). These findings suggest that the interaction between EBV and HLA-DR15 can induce a hyperreactive but defective CD8 T-cell response, as well CD4 T cells cross-reactive with self antigens, both events possibly concurring to MS development (105).

Recent analyses of GWAS data implicate innate and adaptive immunity in MS pathogenesis and several of the non-MHC gene susceptibility variants regulate T cell differentiation and function (6–8). These include variants of the genes encoding: the transcription factors eomesodermin (Eomes, also known as T-box brain 2) and T-bet (T-box expressed in T cells, highly homologous to Eomes) which promote the differentiation and activation of type-1 immunity cells (Th1, Tc1, NK cells) implicated in the defense against intracellular pathogens and viruses (106); IFNγ receptor β-chain, involved in the response to IFN-γ, a major product of activated Th1, NK and CD8 T cells; molecules related to the signaling and production of IL12, a cytokine produced by dendritic cells and macrophages with a crucial role in the commitment to Th1 and Tc1 cells; molecules involved in the production and signaling of IL15, a cytokine essential for the development, homeostasis, function and survival of CD8 T cells; the pore-forming protein perforin, a key component of the lytic machinery of cytotoxic lymphocytes. Other MS associated risk variants that are involved in T cell function encode the following molecules: subunits of the IL2 receptor and IL7 receptor that regulate T cell genesis, survival and expansion; CD28 and CTLA4 expressed on T cells and their ligands CD80 and CD86 expressed on antigen presenting cells (APC), which are major regulators of T cell activation and inhibition, respectively; genes involved in CD28 and CTLA4 signaling; CD40 and CD58, both expressed on APC and implicated in APC-T cell interactions; CD69, an early T cell activation marker; CD161, highly expressed in a subset of CD8 T lymphocytes implicated in MS pathogenesis (see below).

GWAS have also identified MS-risk gene variants that encode molecules associated with B cell development and activation (7, 8). These include: CD40, whose signaling is mimicked by the EBV latent protein LMP1 (11); tumor necrosis factor receptor-associated factor adaptor protein 3 (TRAF3) interacting with both CD40 and LMP1 (107); all members of the Fc receptor-like gene family, primarily expressed on B cells; MYB, a transcription factor required for B-cell development; molecules linked to IL6 and IL10 signaling pathways. *NFKB1* encoding nuclear factor kappa B (NF-κB), a major regulator of the immune response, also emerged among the genes that increase MS susceptibility. Interestingly, the NF-κB pathway is activated in CSF memory B cells from MS patients (108) and a mutation in the NFKB1 gene has been implicated in a primary immunodeficiency characterized by poor control of EBV and lymphoproliferation (109).

A recent GWAS has identified a variant of the *TNFSF13B* gene encoding the cytokine B-cell activating factor (BAFF) involved in B-cell activation, differentiation, and survival, that is more common in Sardinia, an Italian region with one of the highest MS prevalence rates worldwide, and is associated with an increased risk of developing MS (110). The *TNFSF13B* variant (named BAFF-var) leads to increased levels of serum BAFF, immunoglobulins and circulating B cells, particularly memory B cells, the main reservoir for life-long EBV infection, raising the possibility that BAFF-var might favor an altered EBV-immune system balance.

## CD8 T Cell Frequency, Phenotype and Expansion in the CNS OF MS Patients

The first hint that CD8 T cells could play a key role in MS came from immunohistochemical studies assessing the frequency and distribution of different T cell subsets in postmortem brain specimens of patients dying during the chronic phase of MS (111–113). These early studies showed that CD8 T cells outnumber CD4 T cells in white matter lesions, perilesional areas and meninges. More recent studies have confirmed predominance of CD8+ cells among T cells in all types of brain areas with inflammatory cell infiltrates (active, chronic active and inactive white lesions and normal appearing white matter), irrespective of disease course (acute, relapsing-remitting or progressive) and extent of immune infiltration (62, 114–116). The numerical data give already valuable information on the T cell population that preferentially migrates and/or reactivates locally in the MS brain. In fact, the CD4:CD8 ratio in the CNS parenchyma of MS patients (mean of 1:3 to 1:5, range from 1:1 to 1:50) is inverse to the CD4:CD8 ratios in the peripheral blood (2:1) and CSF (4-5:1) of MS patients and healthy controls.

CD8 T cells accumulating in brain tissue of MS patients are mainly effector memory T cells (CD45RA-, CD45RO+, CCR7-) that express the activation marker CD69, the costimulatory molecule CD137 and the apoptosis inducing TNF family member CD95L, and produce IFNγ (62, 114, 117). It is less clear whether CD4 and CD8 T cells producing IL17 and GM-CSF are frequent in MS brain lesions, since the available data are contradictory (114, 118–120). A variable but substantial proportion of CNS-infiltrating CD8 T cells express the lytic enzymes granzyme B and perforin (62, 114, 115, 117), their *in situ* cytotoxic activity being supported by lytic granule polarization, cell surface expression of the degranulation marker CD107a (117, 121) and presence of adjacent cells expressing cleaved caspase 3 (62).

CD8 T cells with a memory phenotype (mainly central memory but also effector memory T cells) are enriched in the CSF of MS patients relatively to the peripheral blood (122–125). CSF effector memory T cells produce predominantly pro-inflammatory cytokines, like IFNγ and TNF, and express granzyme B and perforin (114, 123, 125–127). CSF enrichment of effector memory CD8 T cells is more prominent during early stages of relapse onset MS (125), and increased levels of granzyme B were found in the CSF of MS patients during relapses, suggesting higher CD8 T cell-mediated cytotoxicity (128).

Molecules that are expressed in CSF- and CNS tissue-infiltrating CD8 T cells and have been implicated in memory CD8 T cell migration include: the chemokine receptors CCR5 and CXCR3, that bind CCL5 and CXCL9/10, respectively (123, 129), and are enriched in type-1 immunity cells (106); P-selectin glycoprotein ligand-1 (130); junctional adhesion molecule-like (131); melanoma cell adhesion molecule (132); CD11a, the α chain of the αLβ2 integrin (also known as leukocyte function associated antigen 1) (133); and α4 integrin (114), which is targeted by the MS drug natalizumab preventing lymphocyte entry into the CNS (134).

Preferential activation of CD8 T cells recruited to the CNS is corroborated by several studies showing that CD8 T cells, and to a lesser extent CD4 T cells, proliferate and undergo clonal expansion in the CSF and post-mortem brain tissue of MS patients (80–82, 115, 122, 133, 135). TCR repertoire studies using complementarity-determining region 3 spectratyping have shown that clonally expanded CD8 T cells are present in both MS lesions and normal-appearing white matter (80, 83, 133). The same expanded CD8 T clones were identified in different brain areas, indicating high pervasiveness (83, 133), and in matched biopsy brain, CSF and peripheral blood samples from a few MS patients (81, 133). It was shown that some of the brain-infiltrating CD8 T cell clones persist for several years in the CSF and/or blood of MS patients (81) and that clonal CD8 T cells in the blood show a bias towards a memory phenotype with higher expression of CCR5, CD11a and granzyme B, compared to the non-oligoclonal counterparts (133). Together, these findings suggest a strong, persisting CD8 T cell memory response and ongoing exposure of CD8 T cells to an antigenic stimulus in the periphery and in the CNS. By single-cell RNA sequencing of CSF cells, Beltrán and colleagues (82) recently showed that clonally expanded CD8 T cells can be detected in the CSF of MS discordant monozygotic twins with subclinical neuroinflammation defined by presence of small MRI lesions and CSF alterations, supporting early involvement of activated CD8 T cells in MS immunopathogenesis.

## CD8 T Cells in the Peripheral Blood of MS Patients

Changes in the frequency and function of total CD8 T cells or CD8 T cell subsets in the peripheral blood of MS patients have been reported less consistently and appear to be dependent on disease activity, course and duration. Early studies showed reduced CD8 T cell frequencies during relapses and in the progressive phase of MS (136–138). More recently, Pender and colleagues have shown that a deficiency in effector memory and terminally differentiated CD8 T cells is present already in early MS, persists during chronic disease and is associated with a reduced CD8 T cell response to EBV (57, 139). Of interest, studies performed in different control-MS case cohorts have found that expression of the genes encoding Eomes and T-bet, both associated with MS risk, is lower in the peripheral blood of MS patients than in healthy individuals (140, 141). Lower Eomes and T-bet expression was associated with CD56^dim^ NK cells and CD56+ memory CD4 and CD8 T cells (141). Because Eomes and T-bet play a key role in the differentiation and function of type 1 immunity cells (Th1, Tc1 and NK cells), it has been proposed that their reduced expression in MS could be linked to abnormalities in cytotoxic immunity and defective clearance of EBV (141).

An increase in the percentage of CD8 T cells expressing the activation markers CD26 and CD69 was described in patients with clinically isolated syndrome (CIS), a condition predisposing to MS, and MS patients with radiologically active disease compared to patients with inactive disease and healthy individuals (142). In progressive MS, more elevated frequencies of CD8 T cells producing IL4 and IL10 (143), and of both T-bet+ CD4 and CD8 T cells were reported (144).

## Antigen Recognition by Pathogenic CD8 T Cells in MS

### Self Antigens

Despite decades of intense research to prove the autoimmune etiology of MS, disease-associated autoantigens remain undefined (85, 145). CD4 and CD8 T cells recognizing human myelin protein-derived peptides have been detected in the peripheral blood of both healthy individuals and MS patients (146). However, it is controversial whether T cell reactivity to myelin antigen-derived peptides differs between patients and controls. Some studies have shown that CD8 T cell responses to major myelin proteins [MBP, proteolipid protein (PLP), myelin-associated glycoprotein (MAG), myelin oligodendrocyte glycoprotein (MOG)] (147, 148) or other oligodendrocyte proteins (149) are increased in MS patients compared to healthy subjects whereas other studies report no differences (150–152). However, Sabatino and colleagues (152) found a higher frequency of myelin-specific memory CD8 T cells in the peripheral blood of MS patients compared to controls, suggesting prior exposure to antigen.

Fewer studies have tried to evaluate autoreactivity of intrathecal CD4 and CD8 T cell responses in MS using different experimental approaches. T cells isolated from postmortem brain tissue of a patient with aggressive MS proliferated in response to peptides from different myelin proteins [PLP, MBP, MOG and cyclic nucleotide phosphodiesterase (CNPase)] presented by HLA-DR-matched peripheral blood mononuclear cells (153). Recently, it was reported that short term CD4 and CD8 T cell lines derived from the CSF of patients with CIS and definite MS (154) and from the CSF and brain tissue obtained from chronic MS patients at autopsy (62) showed no reactivity towards several candidate MS associated autoantigens, like myelin (MAG, MBP, MOG, PLP), glial (Kir4.1, S100B) and neuronal (contactin-2, neurofascin) antigens, and alpha B-crystallin presented by autologous EBV-transformed B cell lines or an allogeneic HLA-matched EBV-transformed B cell line stably transduced with these human antigens. Similarly, freshly obtained CSF T cells from MS patients were not activated by autologous dendritic cells pre-loaded with complex candidate autoantigens, like human myelin, brain homogenate, and cell lysates of apoptotically modified oligodendroglial and neuronal cells (50). By *in situ* pentamer binding, MBP-specific CD8 T cells were not detected among the immune cells accumulating in active white matter lesions and meninges in brain tissue from progressive MS cases (63).

Autoreactive CD8 T cells specific for apoptotic T cell-associated self-epitopes and producing IFNγ or IL17 were found at higher frequency in the peripheral blood of MS patients compared to healthy subjects and in the CSF of MS patients, suggesting a possible mechanism for amplification of the local inflammatory response (151). Recently, Planas and colleagues (155) identified a ubiquitous enzyme, guanosine diphosphate (GDP)-L-fucose as a potential MS autoantigen recognized by a Th2-like CD4 cell clone expanded in MS brain lesions and by CSF-infiltrating CD4 Th1-like cells from HLA-DRB3*-positive patients, and showed crossreactivity with an immunodominant MBP peptide and homologous peptides from gut microbiota. Overall, the paucity of studies and the disparate findings obtained do not allow to draw conclusions on the autoreactivity of CNS-infiltrating CD4 and CD8 T cells in MS.

The demonstration that B-cell depletion with anti-CD20 monoclonal antibodies is highly effective in reducing CNS inflammation in relapsing remitting MS (89, 156) has fostered investigations on the pathogenic role of B cells in MS, particularly on their ability to promote T cell activation through antigen presentation and cytokine production. In the peripheral blood of MS patients B cells exhibit abnormal pro-inflammatory cytokine production, which can be induced by TLR9 ligand CpG-DNA or IFNγ, and promote T cell and myeloid cell activation (157, 158). A population of activated Tbet+ CXCR3+ memory B cells has been identified that is enriched in CSF (108), meninges and brain tissue of MS patients, is induced by IFNγ and TLR9 signals and has been linked to EBV load in the peripheral blood of MS patients (159, 160).

**Figure 1:**
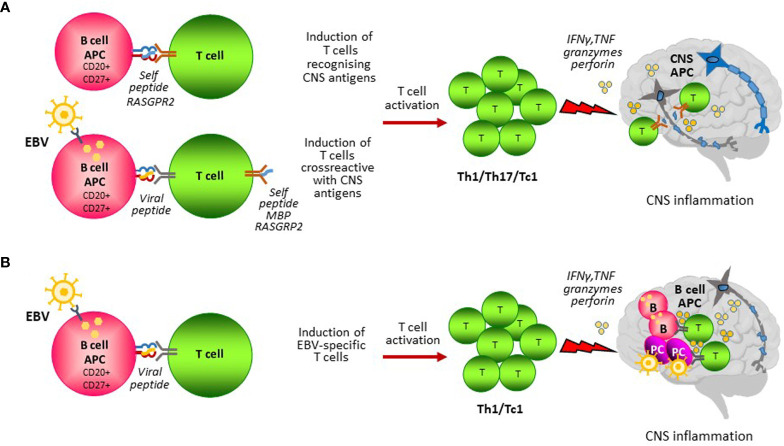
B cell antigen presentation in MS. B cells could contribute to the activation of pathogenic T cells through presentation of self and non-self antigens. **(A)** CD27+ CD20+ memory B cells could present self-peptides from proteins that are expressed in the CNS leading to the induction of autoreactive T cells. Infection of B cells with EBV induces EBV-specific T cells that exert continuous immune surveillance and are essential for virus-host homeostasis. EBV infected B cells could induce autoreactive T cells by presenting EBV peptides sharing similarities with peptides from CNS self-antigens (i.e. MBP, RASGPR2) (molecular mimicry). Autoreactive T cells homing to the CNS would recognize their target antigen on local antigen presenting cells (APC) and become reactivated causing CNS inflammation and tissue injury. **(B)** EBV infected CD27+ CD20+ memory B cells induce EBV-specific T cells that migrate in the CNS to counteract an abnormal EBV infection brought inside the CNS by circulating infected B cells. In this model, B cells would act as APC both in the periphery and in the CNS to stimulate a detrimental antiviral immune response causing CNS inflammation and tissue injury.

A role for B cells as APC capable of stimulating autoreactive T cell responses in MS has been proposed in several studies (**Figure 1A**). Earlier work performed by van Noort and colleagues suggested that EBV-induced expression of the small heat shock protein alpha B-crystallin in B cells and HLA-DR-restricted presentation of this protein may activate autoreactive proinflammatory CD4 T cells that recognize stress-induced alpha B-crystallin in oligodendrocytes and astrocytes in MS lesions (77, 161). A series of studies by Hølmoy and colleagues has shown that B cells from the CSF of MS patients can activate T cells that recognize specific antigenic determinants (idiotopes) from their B cell receptors, suggesting a potential mechanism for intrathecal B cell-T cell interactions promoting CNS inflammation (162). Recently, Jelcic and colleagues (94) have shown that memory B cells from HLADR15+ MS patients activate CD4 T cells in the absence of exogenous antigen and identified a peptide of RAS guanyl-releasing protein 2 (RASGRP2) as a self-peptide responsible for CD4 T cell activation. Because RASGRP2 is also expressed in cortical gray matter neurons (94), it has been proposed that presentation of RASGRP2 by B cells activates CD4 T cells that recognize RASGRP2 in the brain, likely presented by HLA-DR+ CNS APC such as macrophages and microglia. Following this study, Wang and colleagues (72) showed that B cells from HLA-DR15+ MS patients can present self-peptide fragments derived from DR2a and DR2b that activate CD4 T cells and identified memory CD4 T cells in the CSF of MS patients that respond to DR2a or DR2b self-peptides presented by B cells and cross-react with RASGRP2, MBP and peptides from EBV and a commensal gut bacterium. These findings suggest that EBV could trigger potential autoreactive T cells in MS through molecular mimicry between EBV peptides presented by the infected peripheral B cells and self peptides presented by B cells themselves and other APC within the CNS (**Figure 1A**).

Over the years, cross-reactive T cells capable of recognizing EBV and myelin antigens have been identified in several studies. Wucherpfennig and Strominger (73) showed that some HLA-DR15-restricted MBP-specific T-cell clones from MS patients also recognize peptides from EBV DNA polymerase and other viral proteins. CD4 T cells cross-reacting with EBV DNA polymerase and MBP peptides were also detected in the CSF of an individual patient (74). Furthermore, Lünemann and colleagues (75) found that a small percentage of IFNγ-producing CD4 T cells specific for the EBV latent protein EBNA-1 cross-recognize MBP in both MS patients and healthy controls, their frequency being higher in MS patients. Despite these intriguing findings, it remains to be demonstrated that CNS autoreactive CD4 T cells contribute to MS pathology. To date, no data are available on CD8 T cells cross-reactive for candidate myelin antigens and MS-associated pathogens.

### EBV Antigens

EBV itself has long been suspected to be the antigenic driver of the immunopathological response targeting the brain and spinal cord in MS (163, 164), raising the possibility that B cells participate in MS pathogenesis through presentation of highly immunogenic viral antigens. A chronic latent EBV infection and bursts of viral reactivation within the CNS could explain several MS pathological features, such as persistent intrathecal B-cell activation, lesion reactivation and predominance of activated cytotoxic CD8 T cells in CNS immune infiltrates (87) (**Table 1**). However, this issue remains highly debated. On the one hand, several studies have reported complete absence or rarity of EBV infected cells in postmortem CNS tissue from MS patients using PCR-based techniques for EBV DNA or RNA or *in situ* hybridization for the untranslated EBV small RNA (EBER) (62, 165–169). On the other hand, since 2007 our group has repeatedly documented a high frequency of EBV infected B cells and plasma cells in postmortem brain tissue mainly from patients with progressive MS (53, 117, 170–173), but also patients with relapsing-remitting and acute MS (117) and patients with relapsing remitting MS dying because of fatal MS relapses after natalizumab interruption (121, 174). Using EBER *in situ* hybridization and immunohistochemical techniques to detect a large array of EBV latent (EBNA2, LMP1, LMP2A) and lytic proteins (BZLF1, BMRF1, BFRF1, p160, gp350/220), we have provided evidence for a dysregulated, predominantly latent EBV infection in a large proportion (40 to 80%) of CNS-infiltrating B cells, including those forming ectopic B-follicle-like structures in the meninges, and for viral reactivation in up to 20% of plasmablasts/plasma cells present in active WM lesions and at the periphery of meningeal B cell follicles. EBV latent and, less frequently, lytic transcripts were detected in immune infiltrates microdissected from the MS brain (120). Importantly, we have shown that EBV dysregulation in the CNS is characteristic of MS and is not observed in patients with other infectious and non-infectious inflammatory neurological diseases (117, 170), excluding non-specific seeding of EBV infected B cells into the CNS during MS. Abnormal EBV infection in the MS brain was recently reported in independent studies (175–177). Discrepancies across studies may be explained by differences in sample selection and methods or tools to detect EBV (178, 179).

EBV RNA has not been detected in CSF B cells and plasma cells (108, 168) or has been detected in a minority of CSF cell samples from MS patients (180). Small differences in PBMC-associated EBV DNA or RNA levels between MS patients and healthy controls were also reported (49, 180, 181). Altogether, these findings suggest that EBV dysregulation in MS might be predominantly confined to the CNS tissue and, possibly, cervical lymph nodes (172).

In the last 20 years, several studies have examined and compared the T cell response to EBV in the peripheral blood of MS patients and control subjects. With a few exceptions (182, 183), quantitative and qualitative differences in the EBV-specific CD4 and CD8 T cell response, but not in the T cell response to other herpesviruses (typically human cytomegalovirus), have been described for MS patients. Lünemann and colleagues (49) found an increased frequency and broadened epitope specificity of memory CD4 T cells with a Th1 phenotype recognizing the EBV latent protein EBNA-1 in MS patients compared to healthy controls. CD8 T cells recognizing EBV transformed B-cell lines, EBV latent proteins, like EBNA3A and LMP2, or pooled peptides from EBV latent and lytic proteins were found at higher frequency in the peripheral blood of patients with MS compared to healthy controls (23, 54, 184). Expansions of EBV-specific CD4 and CD8 T cells have been associated with active disease at the radiological and clinical level in some studies (53, 55, 56), suggesting a link between activation of anti-EBV immunity and CNS inflammation. However, it has also been reported that EBV-specific CD8 T cells from MS patients display a dysfunctional phenotype, like reduced IFNγ production and cytotoxic activity (55, 58, 185), and that the frequency and functionality of EBV-specific CD4 and CD8 T cells progressively decreases with increasing disease duration (54, 55, 58). A plausible explanation is that T-cell exhaustion during MS is the result of a persistently active, poorly controlled EBV infection.

Presentation of EBV peptides by HLA-E, a non-classical MHC class I molecule engaging CD94/NKG2A (inhibitory receptor) or CD94/NKG2C (activating receptor) on NK cells and the TCR of cytotoxic CD8 T cells, was also investigated in MS patients (186). This study shows increased HLA-E restricted recognition of an EBV lytic protein (BZLF1)-derived peptide by CD8 T cells in MS patients compared to healthy controls, a finding suggesting altered immune control of EBV.

The T cell response towards EBV has been investigated also in the CSF and brain tissue of MS patients. EBNA-1-specific CD4 T cells (51) and CD4 T cells recognizing EBV-infected B lymphoblastoid cell lines (50, 52) have been detected in the CSF of patients with MS and patients with other inflammatory and non-inflammatory neurologic diseases. Instead, CD8 T cells recognizing peptides from EBV latent and lytic proteins, but not CMV proteins, were found at higher frequency in the CSF of patients with MS compared to patients with other inflammatory and non-inflammatory CNS diseases (59). Using high-throughput sequencing of TCR-β chains in CSF and blood, Lossius and colleagues (60) also found selective enrichment of EBV-reactive CD8 T cells mainly recognizing EBV lytic proteins, but not CD8 T cells recognizing influenza A virus, in the CSF of MS patients compared to patients with other inflammatory neurological diseases. Another study showed enhanced CD4 and CD8 T cell reactivity towards autologous EBV-transformed B cell lines in the CSF of patients with clinically isolated syndrome and MS compared to neurological controls and reported selective recognition of EBV lytic proteins by oligoclonal CSF CD8 T cells (61). The reported differences in the intrathecal EBV-specific CD8 T cell response between MS patients and patients with other inflammatory neurological diseases indicate that accumulation of EBV-specific CD8 T cells in the CSF during MS results from an antigen-driven pathologic process and is not the consequence of non-specific recruitment due to ongoing CNS inflammation.

Using T cells recovered from white matter lesions of brain samples obtained from MS patients at autopsy, strong CD8, not CD4 T cell responses were generated towards autologous EBV-infected B lymphoblastoid cell lines, although the precise target antigen was not identified (62). Stainings performed in our laboratory using the *in situ* pentamer technique and HLA class I-matched postmortem brain tissue from progressive MS cases have revealed enrichment of CD8 T cells specific for a broad range of EBV latent and lytic protein-derived peptides, but not CD8 T cells recognizing CMV and influenza A virus peptides, in active white matter lesions and in the meninges (63). It was also shown that EBV-specific CD8 T cells accumulating in the MS brain express membrane CD107a, indicative of a cytotoxic phenotype, and adhere to B cells and EBV infected cells, suggesting specific recognition of their target antigen (63). Although these results support a role for EBV-infected B cells as local APC sustaining a detrimental antiviral immune response (**Figure 1B**), their presence in the MS brain at end-stage disease indicates that the CD8 T cell response fails to get rid of the infection. It is known that EBV adopts a wide range of strategies to compromise both innate and adaptive immunity, including MHC class I and class II downregulation, interference with antigen presentation, and induction of immunosuppressive molecules and immune checkpoint inhibitors (187–189). EBV infected B cells and plasma cells by virtue of abundant expression of latent and/or lytic proteins, viral microRNAs and small noncoding RNAs with immune evasion properties (190) may dispel virus specific T cells which, instead of killing the target cells, would go awry and damage the nearby CNS cells.

Collectively, the few studies exploring anti-EBV immunity in the CNS of MS patients suggest that EBV-specific T cells get access to the cerebral compartment and that skewed intrathecal CD8 T cell responses towards EBV could contribute to CNS inflammation and tissue damage. Future research should aim at better defining the antigenic targets, phenotype, function and evolution of CD4 and CD8 T cell responses to EBV in the blood and in the CNS of MS patients. These studies should help in defining the complex functional versus dysfunctional or exhausted signature of EBV-specific T cells in MS, and verify possible associations with the degree of CNS inflammation which varies by patient, is more prominent at MS onset and decreases as the disease progresses. This information could prove useful for immune monitoring of patients during treatment.

## Cytotoxic CD8 T Cell Interactions in the MS Brain

Although most CD4 and CD8 T cells accumulate within the CNS connective tissues (perivascular space in the neural parenchyma and subarachnoid space in the meninges), some T cells, mainly CD8 T cells in actively demyelinating white matter lesions and at the edge of chronic active white matter lesions, cross the outer glia limitans membrane of the perivascular space and enter the neural parenchyma, suggesting increased tissue invasiveness (111, 112, 114–116).

Under inflammatory conditions, all CNS resident cells (neurons, oligodendrocytes, astrocytes, microglia) can be induced to express MHC class I molecules and present peptides derived from endogenous antigens (self or viral antigens), thus becoming potential targets for CD8 T cells (191–193). Microglia, the myeloid immunocompetent cells of the CNS can also take up, process and cross-present exogenous antigen to CD8 T cells (194). If CNS cells, particularly oligodendrocytes, were directly targeted by CD8 T cells, one would expect to observe intraparenchymal CD8 T cells interacting with and displaying immunological synapses and cytotoxic activity towards these cells. To date, there is scant evidence for such an interaction (195) or for preferential interaction of CD8 T cells with any particular neural cell type in MS brain lesions (62). Rather, in the MS brain CD8 T cells with polarized perforin were shown more consistently to interact with CD163+ mononuclear phagocytes in both the perivascular space and the parenchyma in active lesions (116, 196). CNS-infiltrating dendritic cells contacting CD8 T cells and surrounded by proliferating lymphocytes, most likely CD8 T cells, were also detected within the perivascular cuffs in active MS lesions (135). These findings implicate recruited and local myeloid APC in CD8 T cell reactivation within the MS brain, possibly through phagocytosis of cell debris and antigen cross-presentation.

We have repeatedly visualized CD8 T cells that adhere to, form immunological synapses with and polarize perforin or granzyme B towards B cells, plasma cells and cells expressing EBV lytic proteins in white matter and grey matter lesions and in the inflamed meninges in postmortem brain tissue from progressive MS patients (53, 63, 117, 173). CD8 T cell interactions with B cells or EBV infected cells were also observed in multiple actively demyelinating WM lesions in the brain of relapsing remitting patients who died because of fatal relapses after natalizumab interruption (121, 174). As mentioned above, the visualization of EBV-specific CD8 T cells contacting B cells and EBV-infected cells in the MS brain is highly suggestive of local viral antigen presentation (63).

## CD8 T Cell Activation and CNS Injury

Neurologic deficits in patients with MS are mainly due to demyelination, axonal damage and neuronal loss. Numerous studies have highlighted the key contribution of CD8 T cells to these pathological features. Earlier histopathological studies of post-mortem brain samples from MS patients found that acutely injured or transected axons are more frequent immediately after disease onset as compared to chronic disease stages, and that the extent of axonal damage positively correlates with the number of CNS-infiltrating CD8 T cell and macrophages/microglia (197, 198).

The generation of transgenic mice with focused expression of neoantigens on neurons, oligodendrocytes and astrocytes has allowed to study the detrimental effects of autoimmune responses mediated by cytotoxic CD8 T cells against specific brain cell components (193). *In vitro* studies have shown that neurons can be damaged by CD8 T cells both directly, when induced to express MHC class I molecules and present cognate antigen (199–201), and indirectly, when CD8 T cells are activated following recognition of their cognate antigen on other antigen presenting cells (i.e., oligodendrocytes) and cause collateral bystander damage (202). In models of virus-induced demyelination and viral encephalitis, IFNγ (203–206) and perforin (207) released by activated, virus-specific CD8 T cells are critical for inducing neurodegeneration and demyelination. Likewise, blockade of granzyme B production by CD8 T cells *in vitro* and in the model of experimental autoimmune encephalomyelitis reduces axonal injury and neuronal death (208–210).

IFNγ has a key role in the activation of microglia/macrophages, including activation of macrophage oxidative metabolism and antimicrobial activity (211), and is a potent inducer of NAPDH oxidases (212, 213). Excessive IFNγ production can lead to uncontrolled superoxide generation by microglia/macrophages, which is thought to be the major driving force for demyelination and neurodegeneration in the MS brain (214, 215). Activated CD8 T cells also produce TNF that can cause direct injury to oligodendrocytes and neurons or prevent remyelination (216–218). Collectively, human neuropathological and experimental data support persistent CD8 T cell-mediated cytotoxic activity in combination with a deleterious ‘cytokine storm’ and oxidative stress as major determinants of neural cell damage in MS.

## CD8 T Cell Subsets in MS

Different subpopulations of CD8 T cells have been implicated in MS pathogenesis based on studies in brain tissue, CSF and peripheral blood of patients. The most extensively studied CD8 T cell subsets are CD8 mucosal-associated invariant T (MAIT) cells, CD8 tissue-resident memory (Trm) cells and regulatory CD8 T cells.

Several studies have described MS-associated alterations in a subset of CD8 T cells expressing high levels of the natural killer receptor protein 1a/CD161, producing IL-17 and co-expressing for the large part the semi-invariant Vα7.2 TCR identifying MAIT cells. MAIT cells are a unique innate-like T-cell population that is restricted to the MHC-related protein 1 (MR1) and is mainly activated by bacteria but also by proinflammatory cytokines, like IL12 and IL18 (219). A higher frequency of circulating CD8+ CD161high TCR-Va7.2+ MAIT cells was found in relapsing remitting adult MS (220) and pediatric onset MS (221). Other studies reported a decrease in CD8 MAIT cell frequency in relapsing remitting (222, 223) and primary progressive MS (224, 225) or no differences between MS patients and healthy donors in MAIT frequency, phenotype and activation potential (226). CD8 T cells with MAIT cell-related features were also found in CNS lesions at a seemingly low frequency (220, 223, 226, 227). Despite MAIT-like cells could play a role in exacerbating chronic inflammatory processes due to their ability to migrate into inflamed tissues and produce pro-inflammatory cytokines and lytic enzymes, their role in MS pathology remains obscure. A distinct subset of CD8+ T cells expressing intermediate levels of CD161, with characteristics of effector cytotoxic cells and capable of secreting IFNγ, GM-CSF, IL-17 and IL-22, were recently found to accumulate in the CSF and in brain lesions of MS patients (228).

Tissue-resident memory T cells (Trm) are a T cell subset that, unlike central memory and effector memory T cells, do not recirculate but populate permanently various tissues, including the brain, where they provide a first line of defense against spread of viral infections (229, 230). Trm cells are characterized by high levels of CD103 and CD69, low levels of CD62 and CD27, and rapid production of granzyme B, among several cytotoxic effector molecules. Recently, it was shown that a large proportion of CNS-infiltrating CD8 T cells in postmortem brain samples of patients with progressive MS display the phenotype of Trm cells. Combining the results of different studies the CD8 Trm cells characterized in MS brain lesions are CD69+, CD103+ or CD103-, S1PR1-, CCR7- and CXCR6+ (62, 115, 116). In a study analyzing CSF cells from MS-discordant monozygotic MS twin pairs, clonally expanded CD8 T cells showing characteristics of activated Trm cells were found not only in patients with definite MS but also in the cotwins with prodromal (subclinical) neuroinflammation defined by presence of small MRI lesions and CSF alterations (82). Taken together, these studies indicate that CD8 Trm cells are involved in both the early and chronic stages of MS. Because Trm cells persist at sites of prior viral infection representing an autonomous cytotoxic barrier (231), their presence in the MS brain is compatible with a role in contrasting a persistent and dysregulated EBV infection of B cells accumulating in white matter lesions and in the meninges (53, 117, 120, 170–173). Despite being identified for their protective function, Trm cells can turn into drivers of tissue damage in the context of chronic infections (232).

CD8 T cells have also been identified as potential immunoregulatory cells that can act by direct killing of putative pathogenic T cells and/or through production of immunosuppressive molecules (233). Non cytotoxic CD25+ FoxP3+ CD8 T cells producing TGF-β and IL10 and suppressing autoreactive CD4 T cell activation were identified in the peripheral blood of MS patients and healthy subjects (234). A lower percentage of circulating Foxp3+ CD8 T cells was found in relapsing than in remitting patients with MS and in controls (235). An immunoregulatory role for HLA-E restricted CD8 T cell subsets in MS has also been hypothesized (236). Furthermore, CNS antigen-specific CD8 regulatory T cells with cytolytic activity towards autoreactive CD4 T cells were identified in the peripheral blood of MS patients and their activity was deficient during MS relapse (237). Since the pathogenic effector T cell subsets in MS have not yet been identified, the functional relevance of regulatory deficits in the CD8 T cell compartment remains unclear.

## CD8 T Cells in Other Neuroinflammatory Diseases and Analogies with MS

In addition to MS, CD8 T cells have been implicated in the pathogenesis of several neurological immune-mediated diseases with defined or unknown etiology. Studying and comparing different neurological diseases characterized by prominent CD8 T cell accumulation and activation in the CNS can provide clues about still elusive pathogenic mechanisms and new therapeutic options, such as drugs limiting access of pathogenic CD8 T cells to the CNS.

Rasmussen encephalitis is a rare brain disorder with unknown etiology mainly affecting children; it is characterized by progressive unihemispheric atrophy and drug-resistant epilepsy. In Rasmussen encephalitis, CD8 T cells dominate the brain immune infiltrates and display cytotoxic activity towards neurons and astrocytes (238, 239). Brain-infiltrating CD8 T cells, and to a lesser extent CD4 T cells, undergo clonal expansion and produce IFNγ, TNF and granzyme B (240), suggesting local reactivation. The target antigens of T cells expanding in the brain of patients with Rasmussen encephalitis have not been identified yet, but viral antigens have long been suspected. A recent study combining single cell RNA-seq with TCR Vβ chain sequencing in resected brain tissue suggests that clonal T cells could recognize CMV epitopes (241).

Susac syndrome is a rare neuroinflammatory disease with CNS endotheliopathy affecting mainly young adults (242). In CNS biopsies of patients with Susac syndrome, CD8 T cells dominate the immune infiltrates, adhere to and show cytotoxic activity towards CNS microvessels (243). It has been shown that oligoclonal expansion of terminally differentiated activated cytotoxic CD8 T cells occurs in the CNS of Susac syndrome patients (243) but the antigens driving the immune attack causing endotheliopathy remain unknown.

Cytotoxic CD8 T cells have also been implicated in neuronal cell death in autoantibody-associated encephalitides, particularly those with antibodies to intracellular antigens, which include non-paraneoplastic (like glutamic acid decarboxylase 65 encephalitis) and paraneoplastic conditions (244). Paraneoplastic encephalitides are rare neurological disorders that develop in cancer patients in whom autoimmunity in the nervous system is triggered by ectopic expression of neuronal proteins (such as Hu, Yo and Ma2) in cancer cells. However, no studies have definitively shown which antigens are recognized by CD8 T cells accumulating in the CNS in antibody-associated encephalitides.

In virus-induced encephalitis, like herpes simplex virus encephalitis and cytomegalovirus encephalitis, CD8 T cells dominate the CNS immune infiltrates and the immune attack is directed towards viral antigens presented by the infected neural cells (245). Progressive multifocal leukoencephalopathy (PML) is a CNS demyelinating disease caused by reactivation of JC virus (JCV), a polyomavirus that usually establishes a persistent, asymptomatic infection. PML can develop in immunocompromised patients and in MS patients treated with natalizumab as a consequence of reduced immune surveillance in the CNS (246). The main mechanism of CNS tissue damage is due to lytic infection of oligodendrocytes by JCV. Prominent infiltration of CD8 T cells and clonal expansion of activated CD8 effector T cells specific for JCV large T antigen have been detected in the CNS of patients with MS who develop natalizumab-associated PML (247).

Human T-lymphotropic virus type 1 (HTLV-1)-associated myelopathy or tropical spastic paraparesis (HAM/TSP) is an infrequent complication of HTLV-1 infection causing progressive neurological disability and chronic pain (248). The clinical presentation and pathophysiology of HAM/TSP is similar to the progressive forms of MS. Histopathologically, HAM/TSP is characterized by perivascular lymphocytic infiltration, loss of myelin and axons, and reactive astrocytosis in the spinal cord. HTLV-1 is a retrovirus that infects T cells, mainly CD4 T cells, providing activating and proliferating signals, and is the etiological agent of adult T cell leukemia. In HAM/TSP, circulating, activated HTLV-1-infected CD4 T cells invade the CNS and trigger a cytotoxic immune response towards HTLV-1 antigens presented by the infected CD4 T cells which promotes detrimental CNS inflammation leading to demyelination and neurodegeneration (249). CD8 T cells specific for the dominant HTLV-1 antigen, Tax protein, accumulate in the CSF (250) and in spinal cord lesions of patients with HAM/TSP (251). Within the CNS tissue, Tax-specific CD8 T cells express granzyme B, perforin and IFNγ and contact Tax-expressing HTLV-1-infected cells (251).

**Figure 2:**
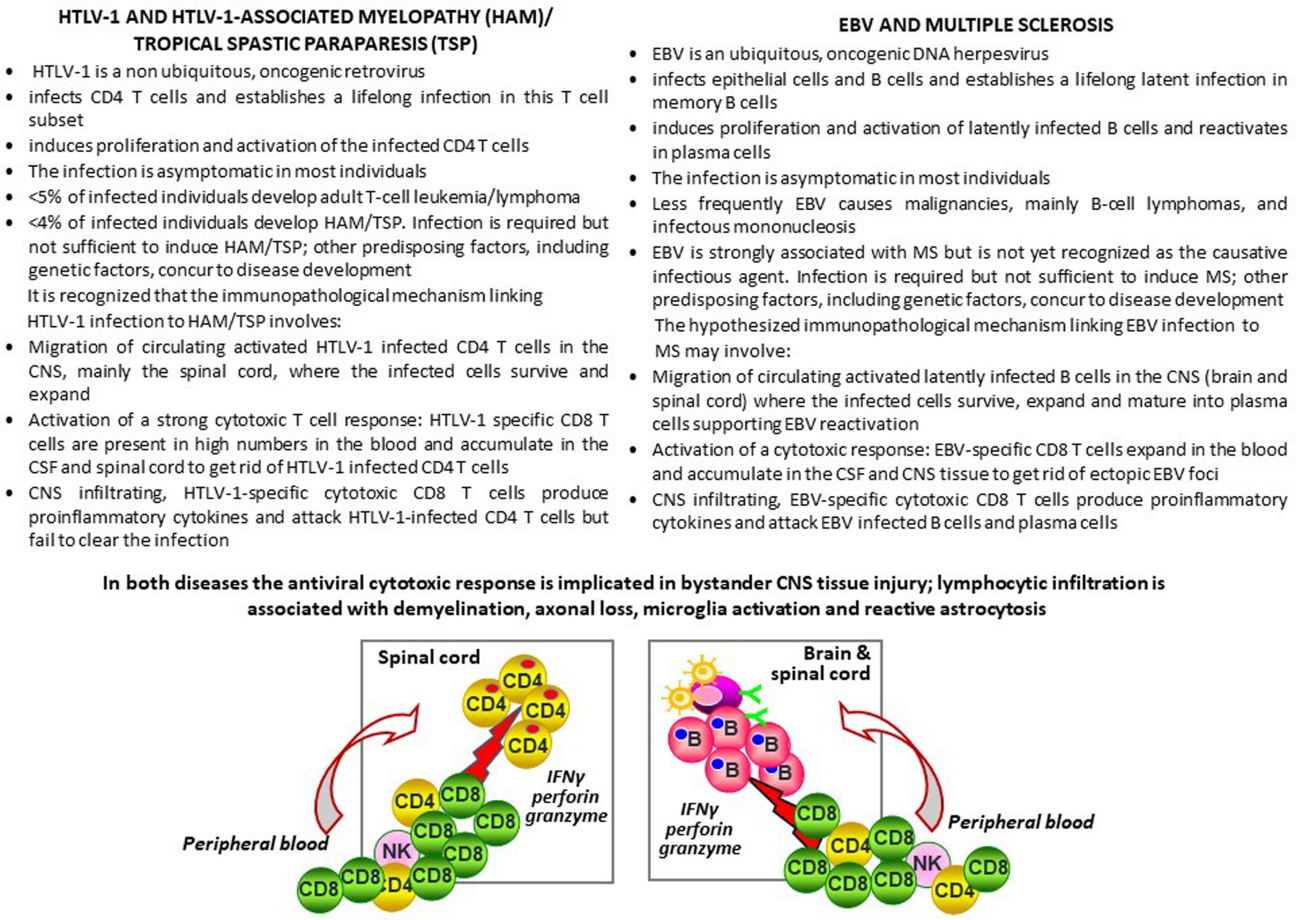
HTLV-1 associated myelopathy/tropical spastic paraparesis and multiple sclerosis: Two chronic CNS inflammatory diseases, two viruses, a common immunopathologic mechanism? The text of this figure summarizes the tropism, biology and pathogenic potential of HTLV-1 and EBV and their association with HAM/TSP and MS, respectively. The HTLV-1-mediated immunopathological model of HAM/TSP is presented vis-a-vis the hypothesized EBV-mediated immunopathological model of MS. The left side of the sketch depicts the migration of HTLV-1-infected CD4 T cells and the activation of a cytotoxic response towards HTLV-1-infected CD4 T cells in the spinal cord in HAM/TSP, leading to production of the pro-inflammatory cytokine IFNγ and the lytic enzymes granzyme B and perforin, which play a key role in bystander tissue injury. On the right side of the sketch, a similar virus-driven immunopathological mechanism involving EBV-infected B cells and EBV-specific CD8 T cells is proposed for MS.

**Figure 3:**
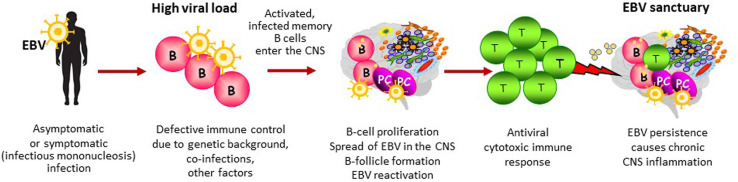
EBV-driven immunopathological model of MS. This figure depicts the main steps potentially leading to establishment of an abnormal EBV infection in the CNS and the ensuing immunopathological response. In individuals at risk of developing MS, defective immune control of the virus could be determined by a high viral load at primary infection (infectious mononucleosis), genetic influences on immune system function, coincident infections and/or any other environmental factor affecting the host’s immune system status. In susceptible individuals, EBV-infected memory B cells could elude immune control and seed into the CNS where they would expand favouring EBV persistence and periodic EBV reactivation (the CNS as an EBV sanctuary). Though activated in the periphery, CNS-homing EBV specific T cells do not clear the virus and become exhausted over time due to persistent, abnormal viral reactivation. The protective antiviral immune response turns into a dysfunctional immune response that promotes CNS inflammation and causes collateral neural cell damage.

HAM/TSP provides an immunopathological model of virus-driven CNS inflammation and bystander neural cell damage that shows several analogies with the postulated EBV-driven immunopathological model of MS (**Figure 2**). Of major interest are the tropism of HTLV-1 and EBV for different lymphocyte populations (T cells and B cells, respectively), their ability to induce proliferation and activation of the infected lymphocytes and exploit their mobility to spread within the host, and the induction of strong cytotoxic responses that counteract the oncogenic potential of both viruses **(** **Figure 2** **)**. To colonize the host, EBV establishes a growth transforming latent infection of B cells and some of the viral proteins expressed during this phase mimic the B-cell activating signals triggered by BCR stimulation and interaction with T cells (11). Activated EBV infected B cells also upregulate adhesion molecules and chemokine receptors that may favor their migration into tissues (159, 252, 253). In infectious mononucleosis, which increases MS risk by about two fold as compared to asymptomatic primary EBV infection, up to 50% of the circulating memory B cells are infected with EBV (254). It is hypothesized that, in a few susceptible individuals, following symptomatic or subclinical primary infection, activated EBV infected B cells are more likely to cross the brain barriers and spread throughout the CNS, similarly to HTLV-1 infected CD4 T cells in HAM/TSP. Establishment of an intrathecal viral infection could be favored by a predisposing genetic background or any other condition affecting permanently or transiently immune surveillance and leading to defective virus control. Due to its particular anatomy, the CNS would hinder complete eradication of the infectious agent, turning into an ‘extralymphatic viral sanctuary’ (255) (**Figure 3**). The reduced ability of cytotoxic T cells to clear the infection would enable the preferential expansion of the infected cells in this organ, thereby explaining the CNS localization and persistence of EBV infected B cells in MS and of HTLV-1 infected CD4 T cells in HAM/TSP.

## CD8 T Cell and B Cell Changes Induced by MS Treatments

Over 15 disease modifying immunotherapies (DMTs) with different efficacy and mechanisms of action are available for the treatment of MS. The newer lymphocyte-targeting drugs are associated with better control of disease activity and long-lasting benefits in MS patients compared to first generation immunomodulatory therapies (interferon beta and glatiramer acetate) (256).

The multitude of treatment options for MS has offered the unique opportunity to get insights into the contribution of different lymphocyte populations to disease pathogenesis. At present, all DMTs approved for MS induce quantitative and/or qualitative changes in T cells but none specifically targets T cells or T cell subsets. Early clinical trials aiming at depleting T cells using anti-CD3 or anti-CD4 monoclonal antibodies did not yield beneficial effects in MS patients. Anti-CD3 monoclonal antibody had major toxic side effects (257). Anti-CD4 monoclonal antibody was less toxic but did not reduce radiological disease activity (258). This result suggests that CD4 T cells are not the best target to reduce CNS inflammation but does not necessarily argue against a role for CD4 T cells in driving CNS inflammation; incomplete CD4 T cell depletion (259) as well as persistence of other pathogenic T cell and APC subsets should be considered as alternative explanations for failure of anti-CD4 therapy in MS. CD8 depleting monoclonal antibodies have been tested in experimental models to block autoimmunity (260), but drugs that target the total CD8 T-cell population are not desirable because lack of specificity would result in enhanced susceptibility to infections. Since the breakthrough finding that the B-cell depleting drug rituximab drastically reduced inflammatory brain lesions and clinical relapses in MS patients (89), B cells began to be considered both as key players in MS pathogenesis and a major target for MS disease control. In the last years, it has become increasingly evident that all DMTs effectively suppressing MS relapses also modulate B cell immunity, leading to focus on B cell-T cell interactions as a potential mechanism driving MS activity (259, 261).

**Table 2** summarizes the effects of licensed DMTs on T and B cells in MS patients. Among low to moderately effective drugs, IFN-β and glatiramer acetate are extensively used as first-line therapies for MS and have broad, still poorly understood immunomodulatory effects. These drugs act mainly by shifting CD4 and CD8 T cells from a pro-inflammatory towards an anti-inflammatory phenotype and by potentiating immune regulatory networks, without affecting significantly T cell numbers. Studies on the effects of IFN-β and glatiramer acetate on B cells are scanty (**Table 2**). Dimethyl fumarate reduces relapses in MS through a still unknown mechanism. This drug causes variable lymphopenia, with partial decrease of circulating memory CD4 T cells, CD8 T cells and B cells, and reduction in CSF leukocyte counts (313). Teriflunomide inhibits pyrimidine synthesis and reduces T cell and B cell proliferation, causing moderate T and B cell depletion in the peripheral blood and CSF (314). Interestingly, a decrease in anti-EBV antibody titers was recently observed in MS patients treated with teriflunomide (315), suggesting potential interference with EBV infection. Consistent with this, it has been shown that teriflunomide inhibits the growth of EBV-transformed B cells and lytic EBV reactivation (316).

**Table 2:** Effects of MS therapies on T cells and B cells in the cerebrospinal fluid and peripheral blood of patients with multiple sclerosis.

Disease modifying immunotherapy	Cerebrospinal fluid	Peripheral blood
IFNβ	Not reported	Reduced frequency of IFNγ producing CD4 and CD8 T cells (262, 263)
		Reduced CXCR3 expression on CD4 and CD8 T cells (264)
		Decrease in CD8 T cells expressing activation markers (CD26, CD71) (142)
		Increase in CD8 T cells producing anti-inflammatory cytokines (IL10, IL13) (265)
		Reduced number of memory B cells and decrease in EBV gene expression (266)
		Absence of CD8 T cell response to EBV in stable MS patients (53)
Glatiramer acetate (GA)	Increase of GA-specific T cells with an anti-inflammatory Th2 phenotype (267)	Induction of Th2 immune responses (268)
		Increase of GA-specific regulatory CD4 and CD8 T cells (269, 270)
		Increased IL10 and reduced proinflammatory cytokine production by B cells (271)
		Increased frequency of EBV latent antigen-specific CD8 T cells, decrease of senescent EBV-specific CD8+ T lymphocytes and memory B cells (272)
Dymethyl fumarate	Reduced leukocyte counts, mainly CD4 T cells (273) and plasmablasts (274).	Variable degree of lymphopenia, with reduction of memory CD4 and CD8 T cells, more marked for CD8 T cells, and memory B cells (273–281)
		Reduced number of pro-inflammatory B cells (278)
Teriflunomide	Not reported	Mild lymphopenia with modest reduction of CD4 T cells, mainly Th1 cells, CD8 T cells (282), and B cells (283)
Fingolimod	Reduced leukocyte counts and lower CD4/CD8 ratio (284); reduced intrathecal B cell clonal expansion (285)	Reduced lymphocyte counts, with marked reduction of T cells, mainly CD4 T cells and the naïve and central memory subsets; B cells, mainly memory B cells, are also reduced (284, 286–289).
Siponimod	Not reported	Reduced lymphocyte counts with marked reduction of B cells, CD4 and CD8 T cells, mainly the naïve and central memory subsets (290)
Ozanimod	Not reported	Reduced lymphocyte counts with marked reduction of B and T cells, mainly naïve and central memory CD4 T cells (291)
Natalizumab	Lower leukocyte counts, reduced numbers of CD4 and CD8 T cells, B cells and plasma cells; lower CD4/CD8 ratio (284, 285, 292, 293)	Higher lymphocyte counts, with increase in effector memory CD4 and CD8 T cells, NK cells and memory B cells (159, 294–296)
		Increased frequency of CD4 and CD8 T cells (297) and B cells (298) with pro-inflammatory phenotype
		Increased frequency of CD8 T cells specific for EBV and other viral antigens (53, 299)
Cladribine	Disappearance of oligoclonal bands in about half of treated patients (300)	Reduced lymphocyte counts with decrease of NK cells, CD4 and CD8 T cells, and more marked and persistent reduction of B cells, mainly the memory subset (301–305)
Alemtuzumab	Not reported	Marked lymphopenia with decrease of all lymphocyte populations, with more persistent depletion of T cells than B cells, hyperpopulation by immature and naïve B cells and marked long term depletion of memory B cells (306–308).
Rituximab ocrelizumab ofatumumab	Significant reduction of B cells and T cells with rituximab treatment (309, 310)	Marked, long-term B cell depletion (156, 309, 311)
		Small reduction of T cells (309) and reduced pro-inflammatory CD4 and CD8 T cell responses (157) in rituximab-treated patients.
		Reduction of pro-inflammatory CD20+ T cells (312) and CD20+ CD8 T cells specific for myelin proteins (152).

Highly effective DMTs for MS aim at blunting the CNS inflammatory milieu by preventing the access of pathogenic lymphocytes to the CNS, inhibiting lymphocyte activation, or eliminating the pathogenic immune repertoire to reset the immune system (317). Fingolimod, siponimod and ozanimod are structural analogues of sphingosine that functionally antagonize sphingosine 1-phosphate (S1P) receptor-1 expressed on lymphocytes inhibiting their egress from secondary lymphoid organs. The therapeutic effect of these oral drugs is likely due to sequestration of pathogenic lymphocytes into secondary lymphoid organs (318) and is associated with a marked decrease in circulating naïve and central memory T cells, memory B cells and CSF leukocytes (**Table 2**). Natalizumab, a humanized monoclonal antibody (mAb) against the α4 subunit of the α4β1 and α4β7 integrins, prevents migration of circulating leukocytes into the CNS thereby excluding entry of pathogenic lymphocytes (134). This is reflected by a significant reduction of both T cells and B cells in the CSF and an increase of all lymphocyte subsets in the peripheral blood, particularly memory effector T cells, NK cells and B cells (**Table 2**). The reestablishment of lymphocyte migration to the CNS after natalizumab or fingolimod discontinuation may lead to a “rebound effect”, with an increase in the number of relapses and substantial disease reactivation on MRI (319). The rebound effect may suggest that the pathogenic T and B cells have accumulated in the periphery during drug treatment and/or that they receive a stronger antigenic stimulus within the CNS environment after treatment interruption. The rare cases of fatal rebounds after natalizumab withdrawal have multiple active lesions and prominent immune infiltrates populated by B cells, CD4 and CD8 T cells in the CNS (320–322). In two MS cases of fatal post-drug rebound without evidence of JC virus infection in the CNS, massive EBV reactivation and presence of CNS infiltrating EBV-specific CD8 T cells were observed, leading to propose that this condition might represent an EBV-associated immune reconstitution inflammatory syndrome (121, 174).

The lymphocyte depleting anti-CD52 monoclonal antibody alemtuzumab and cladribine are administered as single or short treatment cycles and have long term clinical and immunodepleting effects (259). Alemtuzumab targets the cell-surface glycoprotein CD52 and causes profound depletion of CD4, CD8 T cells and B cells in MS patients (323). After treatment, CD4 and CD8 T cell depletion can persist for one year or longer; B cell recovery is more rapid and the repopulating B-cell pool consists mainly of transitional and naive B cells, with a persistent deficiency of memory B cells (306). Cladribine is a synthetic purine nucleoside analogue that predominantly targets lymphocytes, disrupting DNA synthesis and repair and leading to cell death. In MS patients cladribine induces modest CD4 and CD8 T cell depletion and a more marked and persistent reduction in circulating B cells, mainly memory B cells, which could be the main mechanism underlying its efficacy (301, 302). A preliminary study showing disappearance of CSF oligoclonal Ig bands in about half of MS patients 10 years after cladribine treatment suggests that cladribine also depletes B-cells within the CNS (300).

B-cell depleting anti-CD20 monoclonal antibodies are very effective in treating relapsing MS (89, 156, 311) and exhibit some clinical benefits in active primary progressive MS (324, 325). Rituximab (administered off-label for MS treatment), ocrelizumab and ofatumumab selectively target and cause long-lasting depletion of circulating CD20+ B cells, sparing antibody producing plasma cells and very early B cell precursors (326). As observed with alemtuzumab and cladribine, B cell repopulation by transitional and naïve B cells occurs months later after infusion of rituximab while memory B cell depletion is more persistent (312). Circulating T cells are scarcely affected by anti-CD20 monoclonal antibodies (309). However, it has been shown that rituximab causes a significant reduction of both B cells and T cells in the CSF (309, 310) and of pro-inflammatory T cell responses in the peripheral blood of MS patients (157), suggesting that B cells control T cell activation. Although CD20 is a hallmark cell surface marker for B cells, a small fraction of CD4 and CD8 T cells expresses low levels of CD20. CD20+ T cells with a proinflammatory phenotype are increased in the peripheral blood and CSF of MS patients, can be stimulated by myelin antigens and are reduced by anti-CD20 administration (152, 312, 327).

Because circulating CD4 and CD8 T cells are almost completely depleted by alemtuzumab, partially depleted by cladribine and scarcely affected by anti-CD20 monoclonal antibodies, it could be argued that T cell depletion is not a necessary requirement for effective and durable MS control. Conversely, profound and persistent depletion of circulating memory B cells is a common feature associated with the high therapeutic efficacy of these drugs (259). Because memory B cells are the site of EBV persistence, the long-term memory B cell depletion caused by anti-CD20 monoclonal antibodies, cladribine and alemtuzumab implies that these drugs are actually deleting the main B-cell reservoir of EBV. It is worth noting that rituximab is an effective therapeutic option for EBV+ post-transplant lymphoproliferative diseases and is used in combination with chemotherapy drugs to treat EBV+ B lymphomas (328). In MS, B cell depleting drugs could lower the viral load and hence the burden of an EBV-driven immunopathological response. It is of relevance that widespread, active EBV infection in the brain and spinal cord of a patient with primary progressive MS was accompanied by profound EBV deregulation and marked lymphoproliferation in a deep cervical lymph node, but not in a pulmonary lymph node (172). Although preliminary, this finding points to CNS-draining lymph nodes as the primary site where an immunopathological response targeting intracerebral EBV infection is stimulated. Depletion of EBV infected B cells in CNS-draining lymph nodes could explain the high efficacy of anti-CD20 monoclonal antibodies in reducing MS activity despite their low access to the CNS (329).

Specific interference with B cell activation and maturation by targeting the Bruton´s tyrosine kinase (BTK), an enzyme involved in B cell receptor signaling, represents a promising therapeutic approach for MS that may more selectively remove pathogenic B cells (330). BTK inhibitors are small-molecule drugs that enter the CNS and could target also CNS-infiltrating B cells (331). Interestingly, BCR signaling activates EBV lytic infection and some BTK inhibitors are able to block EBV reactivation in B cells, raising the possibility that these drugs may reduce the pool of EBV infected cells by preventing new infection events (332).

In MS, CD8 T cells have attracted attention not only as key pathogenic effector cells and drug targets, but also for their therapeutic potential. Based on the hypothesis that MS results from a defective CD8 T cell control of EBV infection leading to accumulation of EBV-infected autoreactive B cells in the CNS (66), the feasibility and safety of treating progressive MS patients with autologous EBV-specific T cell therapy was recently assessed in a phase I clinical trial (333). Autologous EBV-specific T cell lines were generated by stimulation of patient peripheral mononuclear blood cells with CD8 T cell epitopes from EBV latent proteins (EBNA1, LMP1, LMP2A) and infused to the patients with the aim to eliminate EBV infected cells and restore the EBV-immune system balance. Autologous EBV-specific T cell therapy was well tolerated without serious adverse events and reduction in disability rate and fatigue was reported in some patients (333). However, larger clinical trials must be performed to establish if adoptive immunotherapy targeting EBV infected B cells can ameliorate MS (334).

## Concluding Remarks

A growing body of evidence from epidemiological, genetic, immunological, neuropathological, molecular and clinical studies in MS ties together CD8 T cells, B cells and EBV. The results of the most recent epidemiological studies confirm the link between EBV infection and increased MS risk, reinforcing a causal association (335–339). It is conceivable that the genetic complexity of MS [including 200 autosomal variants outside the MHC locus, one on the X chromosome and 32 MHC alleles affecting MS risk (8)] might reflect the numerous pathways that EBV, a very complex virus with nearly 100 genes encoded by its genome (11), can exploit to alter the virus-host immune system balance and manifest its pathogenicity. Accordingly, MS risk genes are over-represented as target sites for the EBV transcription factor EBNA2, an activator of cellular and viral genes with a key role in the initial phases of EBV infection (340–342). A recent study in humanized animal models (105) stresses the need to investigate in more detail the interactions between specific MS susceptibility genes and immune control of EBV infection that may predispose to disease development (343). Because MHC class II functions as co-receptor for EBV entry into B cells (189), it would be important to assess if MHC class II alleles associated with increased or reduced risk for MS differentially affect the susceptibility of B cells to EBV infection. Along this line, a preliminary study has shown that HLA-DRB1*15:01 acts as a coreceptor for EBV infection of a B cell line (344).

To date, it is still unknown to what extent persistent intrathecal B cell activation in MS is due to B cells that mature and persist in the CNS, migrate from the periphery, or both. Intrathecal B cell activation could be, at least in part, the manifestation of B cell expansion and maturation induced by a persistent EBV infection in the CNS, but this remains to be demonstrated. The presence of proliferating B cells and the high frequency of EBV-infected B cells and plasma cells in the meningeal ectopic B-cell follicles found in chronic MS points in this direction (36, 53, 117, 170, 171). Understanding whether EBV is involved also in abnormal B cell activation outside the CNS, possibly in CNS-draining lymph nodes, and why current MS treatments do not affect intrathecal B cell activation (21), is critical for defining further the mechanism of action of current MS therapies and for developing drugs that more effectively target the ‘hidden’ pathogenic B-cell component.

An important role for CD8 T cells in MS immunopathogenesis is supported by preferential enrichment, expansion and cytotoxic effector phenotype of this T cell population in the CNS. Several studies suggest that EBV could be a major target of the CD8 T cell response in the MS brain (53, 59–63, 117). The identification of CNS-recruited CD8 T cells with a Trm-like phenotype is consistent with local antiviral immune surveillance (62, 82, 115, 116). However, more research is needed to substantiate an EBV-driven immunopathological model of MS. In particular, it is necessary to better characterize both the virological and immunological aspects of EBV infection in MS patients. At the clinical level, cytotoxic immune cell subsets could be valid biomarkers of treatment efficacy, as indicated by a recent study showing that expansion of CD8+ NK cells in the peripheral blood of MS patients is associated with reduced relapse risk (65). It is foreseen that integration of single cell technologies, like single cell transcriptomics and multiparameter spectral flow cytometry, will soon provide a comprehensive and accurate picture of disease relevant immune cell subsets in the CSF and peripheral blood of MS patients, allowing to monitor their frequency and function during treatment.

A model of MS pathogenesis tying together CD8 T cells, B cells and EBV also offers the opportunity of interpreting the broad range of immunological changes underlying the therapeutic effect of the numerous drugs approved for MS. Most MS treatments reducing CNS inflammation and tissue damage have an impact on both T cells and B cells. Although depletion of circulating B cells appears to be a common denominator associated with DMT efficacy in MS (259), it is still impossible to unequivocally link changes in either or both lymphocyte populations to the therapeutic effect. Furthermore, the impact of DMTs on different immune cell subsets in CNS and lymphoid tissues of MS patients remains largely unknown. If presentation of EBV antigens by the infected B cells had a role in MS pathogenesis, it should be assumed that drugs depleting B cells and T cells act by reducing both the antigenic stimulus and the effector arm of the immunopathologic response. Drugs acting selectively on the B cell population would eliminate the primary antigenic drive and consequently prevent the activation of the T cell effector arm. To understand if there is a relationship between EBV status, immune response to EBV and MS disease amelioration after selective B-cell depletion, antiviral cytotoxic immunity should be monitored over time in patients treated with anti-CD20 monoclonal antibodies.

Finally, the concept that MS is a rare neurological complication of a very common infection offers the rationale for clinical trials to test the safety and efficacy of antiviral drugs (345, 346) and EBV-specific adoptive T cell therapy (333, 334) aimed at improving EBV control and restore the EBV-host balance, and for the implementation of a vaccine to reduce the pathogenic potential of EBV (347, 348). It is reasonable to foresee that a better knowledge of CD8 T cell-B cell-EBV interactions in MS patients will translate into a new generation of diagnostic and prognostic biomarkers, antiviral therapeutics and preventive interventions for MS.

## Author Contributions

CV wrote some sections of the manuscript, FA designed, wrote and revised the manuscript. All authors contributed to the article and approved the submitted version.

## Funding

This work has been supported by intramural funding (Ricerca Corrente of Istituto Superiore di Sanità, n. BFC1).

## Conflict of Interest

The authors declare that the research was conducted in the absence of any commercial or financial relationships that could be construed as a potential conflict of interest.
